# The role of eye movements in perceiving vehicle speed and time-to-arrival at the roadside

**DOI:** 10.1038/s41598-021-02412-x

**Published:** 2021-12-02

**Authors:** Jennifer Sudkamp, Mateusz Bocian, David Souto

**Affiliations:** 1 Department of Neuroscience, Psychology and Behaviour, College of Medicine, Biological Sciences and Psychology, University of Leicester, University Road, Leicester, LE1 7RH UK; 2 School of Engineering, College of Science and Engineering, University of Leicester, University Road, Leicester, LE1 7RH UK

**Keywords:** Psychology, Sensory processing, Visual system

## Abstract

To avoid collisions, pedestrians depend on their ability to perceive and interpret the visual motion of other road users. Eye movements influence motion perception, yet pedestrians’ gaze behavior has been little investigated. In the present study, we ask whether observers sample visual information differently when making two types of judgements based on the same virtual road-crossing scenario and to which extent spontaneous gaze behavior affects those judgements. Participants performed in succession a speed and a time-to-arrival two-interval discrimination task on the same simple traffic scenario—a car approaching at a constant speed (varying from 10 to 90 km/h) on a single-lane road. On average, observers were able to discriminate vehicle speeds of around 18 km/h and times-to-arrival of 0.7 s. In both tasks, observers placed their gaze closely towards the center of the vehicle’s front plane while pursuing the vehicle. Other areas of the visual scene were sampled infrequently. No differences were found in the average gaze behavior between the two tasks and a pattern classifier (Support Vector Machine), trained on trial-level gaze patterns, failed to reliably classify the task from the spontaneous eye movements it elicited. Saccadic gaze behavior could predict time-to-arrival discrimination performance, demonstrating the relevance of gaze behavior for perceptual sensitivity in road-crossing.

## Introduction

The decisions pedestrians make in negotiating their way across traffic rely on sensory, perceptual and cognitive factors. Those factors are less well understood in pedestrians^1^ compared to drivers^2^. To avoid collisions, e.g., when crossing a road, the decoding and prediction of motion trajectories of other road users are of particular importance. The way in which visual information is sampled may thereby play an important role for perceptual judgements.

The influence of eye movements on the perceived motion of abstract stimuli (e.g., simple geometric shapes) has been well-documented. For example, observers perceive target speeds to be slower and are less sensitive in discriminating speed when their eyes pursue a moving target compared to when their eyes remain stationary^3–6^. The direction of corrective saccades performed during pursuit influences perceived speed, with saccades congruent to the motion direction of a pursued target increasing the perceived speed and saccades towards the opposite direction decreasing the perceived speed^7^. Smooth pursuit eye movements facilitate time-to-arrival judgements resulting in a higher accuracy of estimates when observers pursue the approaching object compared to fixating it^8,9^. Similarly, observers are more successful at predicting collisions, i.e., discriminating whether an object would hit or pass a target line, during pursuit compared to fixation independently of whether the target line serves as the fixation location and the object moves towards it or the object serves as the fixation location and the target line moves towards it^10^. When predicting the time-to-arrival of a target, observers are found to perform spontaneous smooth pursuit eye movements followed by a saccade towards the arrival location after the moving object is occluded^11^. However, a functional relationship between the execution of these saccades and task performance could not be determined in that study. On the other hand, saccade execution is associated with both a compression of the perceived visual space^12^ and a compression of the perceived time between visual events^13^. It is thus suggested that saccadic behavior could as well affect the prediction of time-to-arrival, although this has not been investigated using an eye tracking methodology^14^.

The aforementioned findings may be relevant in natural tasks including navigation in traffic. Although they suggest that an unfavorable visual sampling strategy can affect road user safety by introducing perceptual biases and promoting risky crossing decisions based on inaccurate motion estimates, little research has yet explicitly addressed the question of how pedestrians sample visual information and how eye movements influence perceptual judgments in a road-crossing scenario. Traffic safety research assessing gaze during driving can give an indication as to which eye movement parameters may play a role in avoiding collisions and how gaze behavior influences road user perception. A driving simulator study explored spontaneous gaze behavior during collision judgements^15^. Similar to what is reported for time-to-arrival prediction using abstract stimuli^11^, drivers are found to perform a series of saccades between the approaching vehicle and intersection. However, since the drivers need to integrate both the motion of the other vehicle and their own motion towards the intersection to solve a collision judgement task, it remains difficult to disentangle whether the observed gaze patterns serve the purpose of estimating their own time-to-arrival at the crossing point or the time-to-arrival of the approaching vehicle. A series of recent studies investigated the effects of gaze location on drivers’ speed estimates of approaching vehicles at an intersection^16,17^. In a two-interval forced choice task, observers judge the speed to be lower when they fixate towards the centroid of an approaching vehicle as compared to its front. Moreover, the size of the approaching vehicle influences how observers spontaneously sample visual information as indicated by a tendency of observers to place gaze towards the front of smaller sized vehicles and towards the centroid of larger sized vehicles. The authors suggest that different visual sampling strategies used by observers when estimating vehicle speed may be partly responsible for perceptual biases such as the size-speed illusion, i.e., larger vehicles appear slower in their approach^18^. This conclusion is particularly interesting with regards to the effect of size depending on the type of estimate being performed. While larger objects appear to move slower, they also appear to arrive earlier, an effect termed the size-arrival effect^19^. When directly comparing both size effects in a traffic scenario simulating a driver’s view at an intersection, an effect of vehicle size is only found on speed but not on time-to-arrival judgments^20^. If the perceptual bias on the perceived speed originates in gaze behavior, this may imply that spontaneous gaze behavior differs between the two judgements.

While research on driving behavior can provide some important insights into the connection of gaze behavior and road user perception, it remains unclear how pedestrians use and sample visual information when evaluating whether the approaching traffic allows them to cross safely. One important aspect is the relevance of various motion parameters for pedestrians’ evaluation of the approaching traffic. For example, while pedestrians’ crossing decisions correlate highly with the time-to-arrival of vehicles at the crossing point^21,22^, other parameters such as vehicle speed and distance have been shown to both bias time-to-arrival estimates and affect crossing decisions^23,24,25^. Another important aspect concerns where pedestrians pay attention to evaluate those parameters. Exploring eye movements in a simple road-crossing scenario could help bringing some clarification to these questions. Here, we specifically focus on two commonly considered motion parameters for road user interactions, i.e., the speed and time-to-arrival of vehicles. We assessed spontaneous gaze behavior during speed and time-to-arrival judgements to explore how eye movements influence task performance in a virtual road-crossing scenario. This allowed us as well to address the question of which visual cues observers attend to in order to evaluate the different motion parameters of the approaching traffic. Moreover, if eye movements were reflective of which kind of motion parameter an observer evaluates, they could as well provide some information about the perceptual demands pedestrians experience during crossing decision-making. There have been long-standing attempts to deduce task demands from eye movements. For example, eye movements could successfully discriminate between free viewing and visual search^26^ as well as between visual search and memorization^27,28^, supporting the idea that cognitive and perceptual processes can be decoded from gaze behavior^29^. In motion perception, however, there is little evidence on whether the evaluation of different motion parameters is reflected in distinctive gaze patterns. Complementary to the goal of testing the functional relationship between eye movements and perceptual estimates, we tested to which extent basic eye movement metrics could predict the perceptual task.

In the present study, we first address the question of how observers use and sample visual information in a road-crossing scenario and whether gaze behavior differs depending on whether observers are asked to discriminate vehicle speed or time-to-arrival. We secondly explore to which extent eye movements are predictive of the discrimination task by training and testing a classification algorithm to discriminate the tasks based on gaze patterns. To do so, we presented participants with video sequences of a simulated road scene displaying an approaching vehicle varying in speed and time-to-arrival as viewed from a pedestrian’s point of view at a zebra crossing. After a fixed display interval, the vehicle disappeared. Each discrimination was performed within a two-interval forced choice task prompting participants to either identify the interval displaying the faster vehicle (speed discrimination task) or the interval displaying the vehicle that would have arrived earlier at the road crossing (time-to-arrival discrimination task). This allowed us to use tasks that differed only in regard to the motion parameter participants were asked to evaluate. Additionally, it allowed us to assess discrimination sensitivities in a simulated but more naturalistic task scenario compared to the stimuli commonly employed in basic vision studies. Thirdly, we address the question of how the previously described effects of eye movements on speed and time-to-arrival judgements generalize to a road-crossing scenario by investigating the influence of gaze behavior on discrimination performance.

Here, we focus on the upper limits in acquiring information about the speed and time-to-arrival of vehicles under ideal conditions in terms of visual sampling (the scenes provided clear visibility and were visually uncrowded), predictability (vehicles approached at a constant speed and disappeared from sight after a fixed display interval) and cognitive demands (there was no competition for cognitive resources, such as when monitoring vehicles on multiple lanes, following a conversation or texting). For this reason, no elements other than the car or the road were simulated. Those idealized and well-controlled conditions enable a first step to be taken towards understanding the determinants of pedestrian behavior.

## Methods

### Observers

Fifteen participants (9 women, 18–43 years old, mean age 27.7 $\pm$ 7.3 years), including the authors JS and DS, took part in the experiment. All participants had normal or corrected-to-normal vision and their visual acuity was verified prior to the experiment. The study was conducted in accordance with the principles of the Declaration of Helsinki and approved by the University of Leicester’s ethical committee. Written informed consent was sought from all participants prior to the experiment.

### Apparatus

Stimuli were displayed on a CRT monitor (HP P1130 CRT) with a 1280 by 1024 pixels screen resolution and 85 Hz refresh rate. We used the Psychophysics Toolbox Version 3 (PTB-3^30^) in Matlab to display pre-generated videos of a simulated road scene created in Unity3D 2018.3.1f1. The videos had a 1280 × 1024 resolution and were displayed full screen, corresponding to approximately 30 × 25 degrees of visual angle. A chin rest was used to stabilize participants’ head at a 66 cm distance from the screen. Eye movements were recorded using an Eyelink 1000 eye tracker^31^ at a 1000 Hz sampling rate. Participants responded by pressing one of two buttons corresponding to the selected interval on a keyboard. Eye movement and response data were pre-processed in Matlab R2018b^32^ and statistical analyses were performed in the R software environment^33^ using the *e1071* package for training and testing the Support Vector Machines^34^, the *lme4* package for generalized linear mixed model analyses^35^ and the *dominanceanalysis* package for dominance analyses^36^.

### Design and procedure

**Figure 1:**
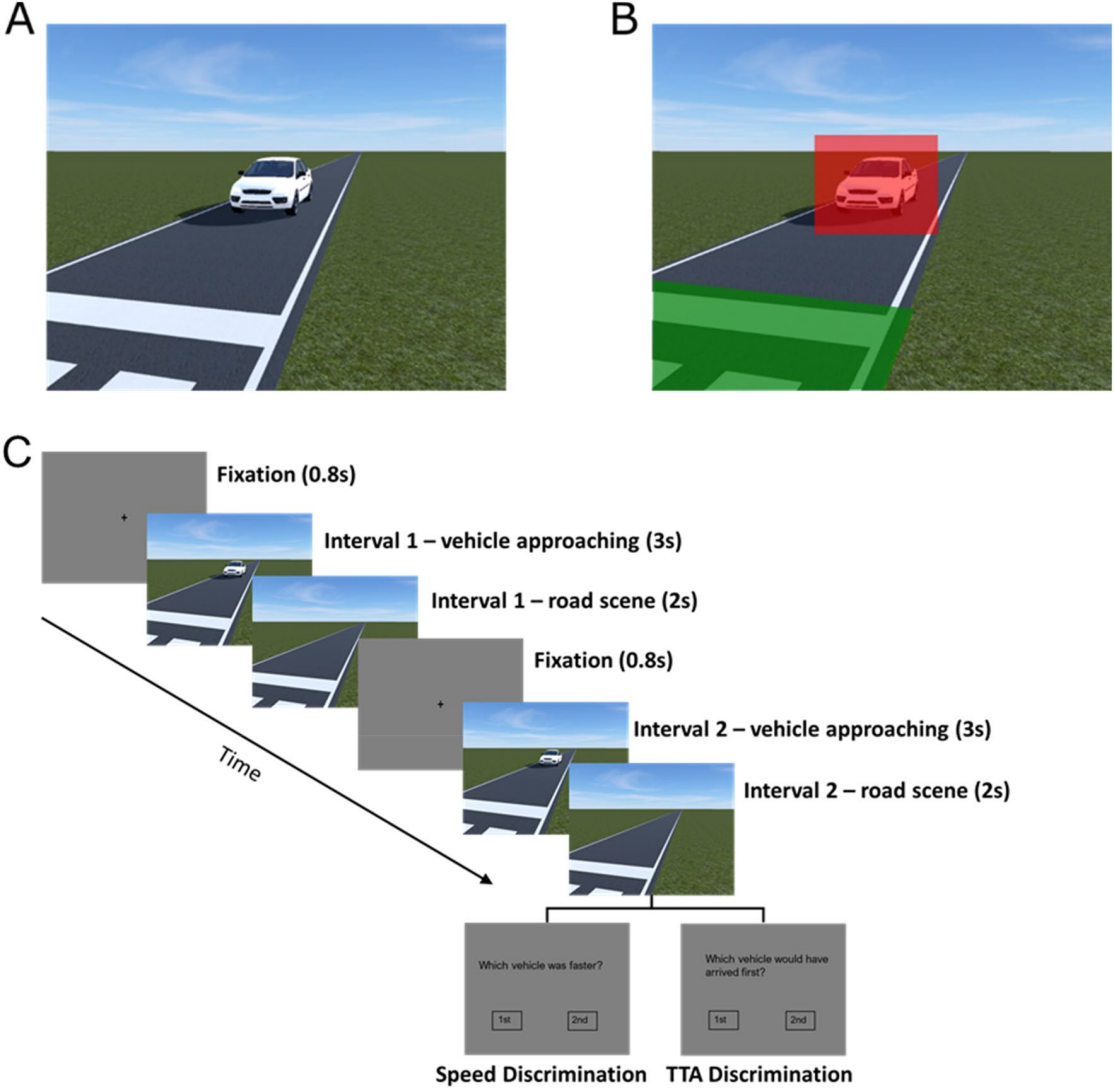
Sample image from the video sequences used in the experiment. (**A**) Shows the visual scene as seen by the participants. (**B**) Shows the Areas of Interest (AOIs) of the road crossing area (green) and vehicle (red) overlaying the visual scene. The center of the vehicle AOI was used to calculate the horizontal and vertical gaze position deviation. (**C**) Shows a trial sequence. Display times in seconds are indicated in brackets.

The video sequences depicted a white car approaching a road crossing area at a constant speed on a single-lane rural road with a virtual width of 3.36 m. The vehicle resembled a Ford Focus Sedan model with approximate dimensions of 4.53 × 1.82 × 1.47 m. The simulated eye height of the observer was 1.6 m and the distance between the observer and the curb was 0.64 m. Figure 1A shows a sample image from the video sequences used in the experiment.

Using a within-subjects design, participants completed the speed and the time-to-arrival discrimination task in two separate sessions. The task order was counterbalanced between participants. Each discrimination was based on a two-interval forced choice task prompting participant to either identify the interval displaying the faster vehicle (speed discrimination) or the interval displaying the vehicle that would arrive earlier (time-to-arrival discrimination). Each of the two intervals was preceded by a black fixation dot, which was displayed for 0.8 s at the center of the screen. The simulated vehicle then approached the road-crossing area for 3 s before the vehicle disappeared and the road scene remained visible for an additional 2 s. After viewing both intervals and indicating which interval contained the faster vehicle or the vehicle that would have arrived first, participants received auditory and visual feedback about the correctness of their response. Figure 1C shows a trial sequence and a demonstration of the tasks can also be foundonline (10.25392/leicester.data.12861119.v1).

**Figure 2:**
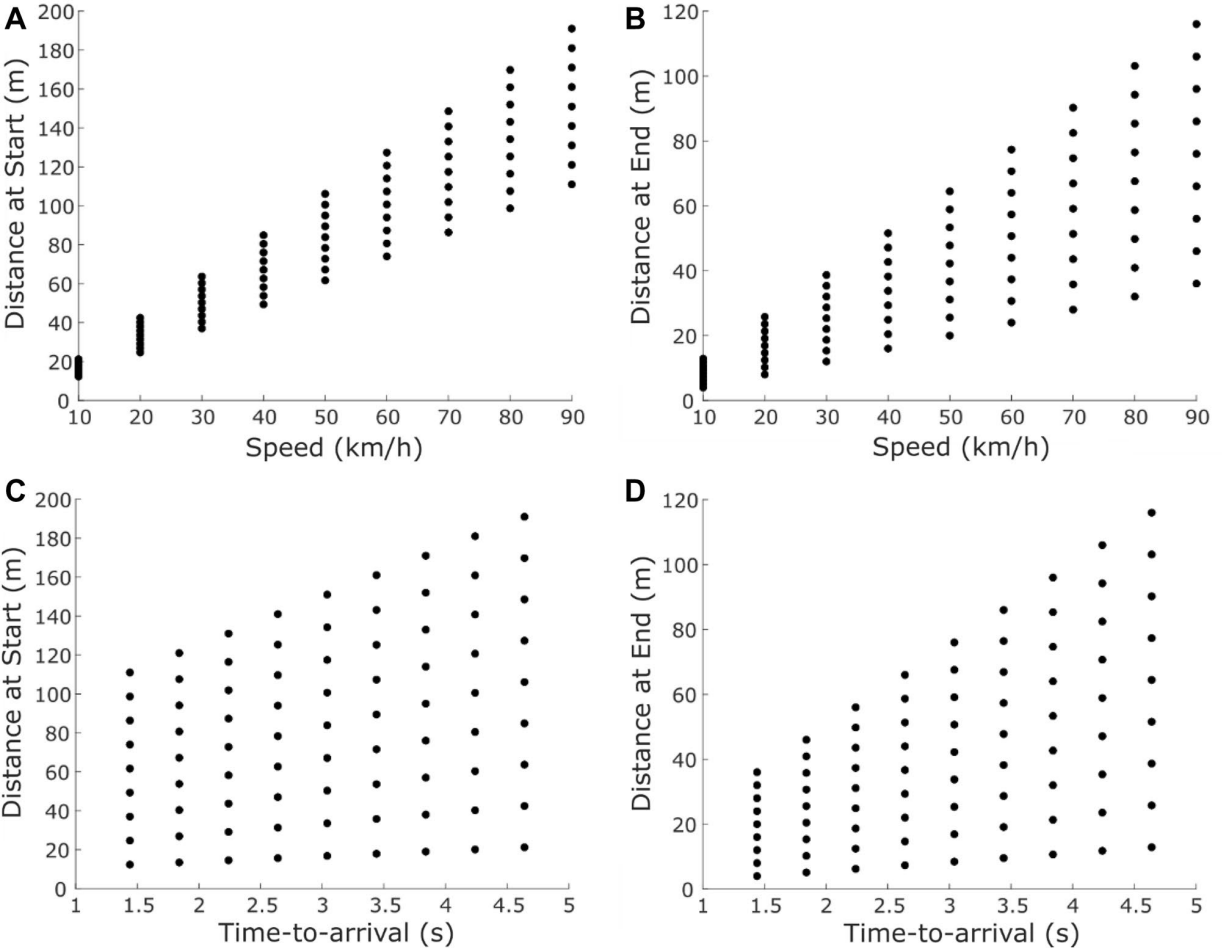
Distribution of the start and end position of the vehicle across speed and time-to-arrival levels. Start and end position were measured as the distance between the front edge of the vehicle and the stopping line in the virtual world in meters (m). Upper panels show the association between vehicle speed, start distance (**A**) and end distance (**B**). Lower panels show the association between the time-to-arrival, start distance (**C**) and end distance (**D**).

In the standard interval, the vehicle approached the road crossing area with a constant driving speed of 50 km/h and had a remaining time-to-arrival at the stopping line of 3.04 s when it disappeared. For both tasks, we used the same 81 unique variations of the comparison interval, which resulted from the combination of nine vehicle speed levels (10, 20, 30, 40, 50, 60, 70, 80, 90 km/h) and nine time-to-arrival levels (1.44, 1.84, 2.24, 2.64, 3.04, 3.44, 3.84, 4.24, 4.64 s). Since we employed a fixed display time, the start position and the end position the vehicle reached before disappearing necessarily varied as a function of its speed and time-to-arrival, resulting in an association between speed, starting distance (Fig. 2A) and end distance (Fig. 2B) as well as between time-to-arrival, starting distance (Fig. 2C) and end distance (Fig. 2D).

In each task, participants viewed three test blocks displaying all 81 speed and time-to-arrival combinations in randomized order, resulting in a total of 243 trials per session. The presentation order of the standard and comparison interval was randomized between trials. The direction from which the two vehicles approached (as in Fig. 1A or its mirror image flipped around the vertical axis) was alternated between trials, but both the standard and comparison vehicle always approached from the same direction.

### Data analysis

#### Perceptual judgements

We fitted two cumulative normal functions to each participant’s response data using a maximum likelihood method implemented by the Palamedes toolbox^37^ in Matlab. The first function modeled the relation between vehicle speed and the proportion of judgements indicating that the comparison vehicle was perceived as being faster (“faster” judgements). The second function modeled the relation between the time-to-arrival and the proportion of judgements indicating that the comparison vehicle was perceived as arriving earlier (“earlier” judgments). The slope, point of subjective equality (PSE; between the minimal and maximal stimulus value), lapse rate (between 0 and 0.1) and guess rates (between 0 and 0.05) were free to vary and were fitted separately for each participant. Our primary interest was to estimate the just-noticeable differences (JNDs), i.e., the differences in the speed and time-to-arrival of the vehicles that participants could reliably discriminate. We calculated the JNDs for each participant and task as half the difference between the value that generated 75% and 25% “faster” or “earlier” responses. The standard errors of the estimated JNDs as well as each functions’ deviance, a statistic that relates to the likelihood of the model fit relative to a saturated model fit^38^, were used to assess the goodness-of-fit of the psychometric functions^39^. Weber fractions were calculated by dividing the JNDs by the respective standard speed (50 km/h) and time-to-arrival (3.04 s).

Since there was no manipulation implemented that distinguished the standard and comparison interval stimulus other than its speed and time-to-arrival, comparison vehicles driving at 50 km/h or having a remaining time-to-arrival of 3.04 s should theoretically yield 50% of responses “faster” or “earlier” as they were identical to the standard in this case. To examine for response biases, we extracted the PSEs from each participant’s psychometric function to test this assumption. We further investigated the presence of an interval bias, which is often observed in two-interval forced choice tasks^39^ by fitting a psychometric function to the proportion of times the vehicle in the *second* interval was judged to move faster or to arrive earlier in relation to its speed or time-to-arrival. An interval bias would be reflected in a shift of the PSE, indicating a propensity to choose one interval over the other.

To explore participants’ use of motion and position cues, we performed logistic regressions to predict each participant’s “faster” and “earlier” responses by vehicle speed, time-to-arrival, start and end position. Subsequently, we conducted dominance analyses to assess each predictor’s general dominance, i.e., the average additional variance explained by an individual factor pitted against all other combinations of factors, to determine the relative predictor importance^40,41^.

#### Eye movements

For all analyses reported herein we used the gaze data recorded during the comparison interval. We selected this interval as we assumed that participants could internalize the motion of the standard vehicle during the course of the experiment, resulting in gaze behavior during the standard interval being less indicative of performance. On the other hand, we expected a higher trial-by-trial variation in participants’ gaze behavior due to the different comparison vehicle trajectories, which would make it more difficult to detect overarching differences between perceptual tasks. We therefore repeated all analyses with gaze data recorded during the standard interval. The comparison of gaze behavior between the two tasks yielded similar results for both the standard and comparison interval. In terms of the effect of gaze on performance, we found slightly different effects for the standard interval, which we discuss briefly and report in further detail in the supplementary material.

To identify blinks and saccades in the gaze data we used the Eyelink event detection algorithm implemented in the EyeLink 1000 Host Application software^31^ with a velocity threshold of 22$^\circ$/s and an acceleration threshold of 5000$^\circ$/s^2^. A moving average of gaze velocity over the last 40 ms is added to the velocity threshold to account for smooth pursuit eye movements in the saccade detection (fixup limit = 60°/s). Eye movements were filtered for blinks and saccades and the removed data were interpolated linearly. Trials were excluded when the interpolated data exceeded 50% of all samples in either interval. One participant was excluded from the eye movement analysis due to the number of excluded trials exceeding 50% of all trials in the speed condition. Across the remaining participants 429 trials (6% of all trials) were removed.

We derived the following eye movement measures from the eye movement recordings using custom Matlab scripts: pursuit gain, relative and absolute horizontal and vertical gaze position deviation, number and average amplitude of saccades and number of saccades targeted towards the road crossing area (before and after the vehicle disappeared). Pursuit gain describes the angular gaze velocity relative to the angular velocity of the pursued target. A gain > 1 signifies that gaze was faster than the vehicle, a gain < 1 signifies that gaze was slower. For the computation of pursuit gain we calculated gaze and target velocities by deriving angular velocities based on the cross product of consecutive 3-dimensional gaze and target vectors in a world reference frame^42^. Although originally aimed at providing gaze estimates in a 3D virtual environment, we adopted this procedure for the present study as it allows for a more precise approximation of angular velocity compared to the commonly used approach of assuming the angular velocity of a stimulus projected on the screen is the same for different gaze-angles relative to the screen. The resulting gaze velocity profiles were filtered using a low-pass, second-order Butterworth filter with a cut off frequency of 10 Hz before entering them in the pursuit gain computation. The first saccade after vehicle onset was removed before counting the total number of saccades and deriving the average amplitude. To determine the number of saccades towards the road crossing area and the deviation of participants’ gaze position from the position of the car, two areas of interest (AOIs) were defined (see Fig. 1B). One AOI covered the road crossing area including the pedestrian crossing and stopping line. For the second AOI a rectangle was defined, which spaciously covered the visual borders of the vehicle. The size of the vehicle AOI increased dynamically according to the increasing size of the displayed car during approach. The center of the vehicle AOI was used to calculate the average horizontal and vertical gaze position deviation in visual degrees. The absolute deviation describes the vertical and horizontal deviation of gaze from the AOI center independent of its direction. The relative deviation includes the directional information with a positive deviation indicating that gaze was placed above (relative vertical deviation) or ahead of the AOI center (relative horizontal deviation). Pursuit gain as well as relative and absolute position deviations were averaged for the 2–3 s time interval before the car disappeared. We chose this temporal window because the retinal speeds of the approaching vehicles were too slow to allow for an accurate measurement of pursuit gain in earlier intervals when the vehicles were still at a greater distance.

Outliers in the gaze data of each observer were detected using a median absolute distance criterion (MAD)^43^ and data points were removed if they exceeded a cut-off value of 3.5 in the case of symmetric distributions and by using a double-MAD criterion with a cut-off value of 4.5 when distributions were asymmetric, as it was the case for the number and amplitude of saccades. On average, 4.27% (min = 3.08%, max = 7.26%) of data points per observer were removed.

To compare the eye movement measures between conditions we performed a repeated measures MANOVA on the averaged gaze data of each task. Moreover, we used the trial-by-trial eye movement data to train a Support Vector Machine (SVM) to predict the perceptual task for each participant based on gaze behavior. The objective of an SVM classifier is to find decision boundaries in the values of the given features that discriminate between the classes (here perceptual tasks) they originate from. The defined decision boundaries are then employed to predict the class affiliation of new data points. We employed a cross-subject validation procedure: in each of the 14 iterations, the data of one participant were set aside and the classifier was trained on the remaining participants. The classifier was then tested on the omitted participant and the procedure was repeated until each participant served as a test. To test for idiosyncratic gaze patterns, we trained a second classifier to predict the observer using a tenfold cross validation procedure, in which each iteration randomly set aside 10% of the data to be used as the test set^44^. To evaluate the performance of the classifiers, we derived the average accuracies of predictions (across participants and folds) and compared them to the no information rate, i.e., the accuracy of predictions achieved by always predicting the class with the highest number of data points in the test set, using binomial testing^45^. We further performed permutation tests to compare the average accuracy of predictions against the null hypotheses that the data and class labels are independent. Permutation-based *p*-values based on 1000 random permutations for each of the two class labels (perceptual task and observer) were calculated by dividing the sum of accuracies gained from the randomized datasets that were equal or higher than the average accuracies gained from the original dataset by the number of permutations^46,47^.

To investigate the influence of gaze behavior on discrimination performance, the trial-by-trial eye movement measures were entered as predictors in a hierarchical logistic regression model predicting the proportion of correct responses in each task.

The study was not pre-registered. The data and materials can be accessed on the OSF project website (https://osf.io/tdkr3/).

## Results

In two separate sessions, participants discriminated the speed and time-to-arrival of approaching vehicles. We measured participants’ gaze behavior during both conditions and investigated its effects on discrimination performance.

### Speed and time-to-arrival discrimination

**Figure 3:**
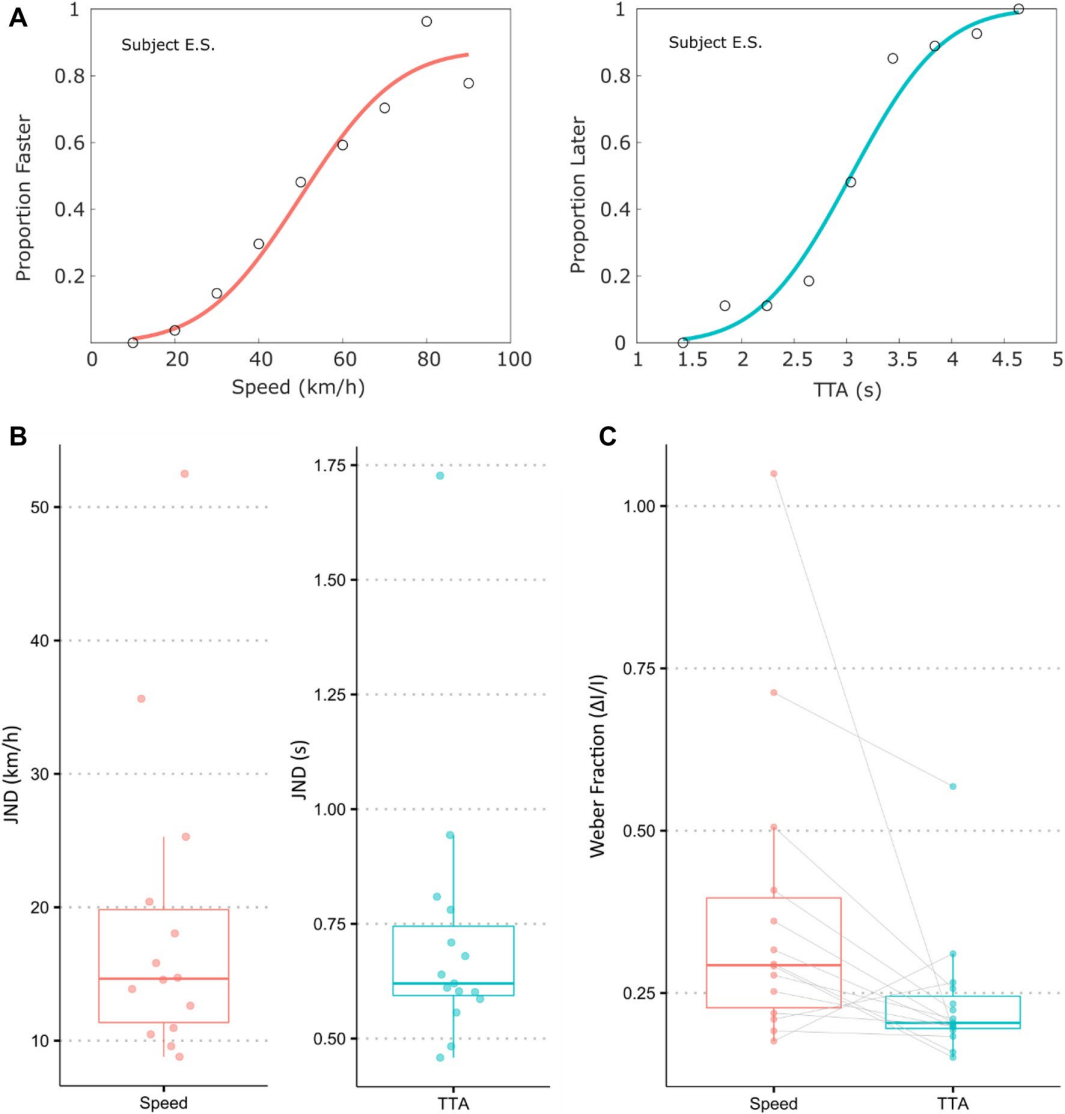
Example of psychometric functions of one participant, JNDs and Weber fractions. (**A**) The left panel shows the proportion of trials the exemplar participant judged the comparison vehicle as faster than the standard vehicle as a function of vehicle speed. The right panel shows the proportion of trials the exemplar participant judged the comparison vehicle as arriving later as a function of vehicle time-to-arrival. The proportion of “later” responses was calculated from the participant’s “earlier” responses and plotted to ease comparison. (**B**) Shows JNDs of all participants in the speed discrimination task (left panel; *N* = 14) and time-to-arrival discrimination task (right panel; *N* = 15). (**C**) Shows the Weber fractions for speed and time-to-arrival discrimination. Crossbars indicate median values. Whiskers depict the 1.5 × interquartile ranges.

Figure 3A shows the psychometric functions fitted to the response data of one exemplar participant. In the speed discrimination task, the fitted model of one participant deviated significantly from the saturated model (*Dev* = 30.81, *p* < 0.001). We therefore excluded the data of this participant from further analyses of the speed discrimination responses. For the remaining participants, the standard errors of the JND and deviances indicated good fits (mean *SE* = 3.72 km/h, min = 1.48, max = 17.95; mean *Dev* = 8.07, min = 2.43, max = 13.83, all *p* > 0.060). The estimation of JNDs in the speed discrimination task (see Fig. 3B) indicated that on average participants were able to discriminate speed differences of 18.08 km/h (*SD* = 12.05 km/h).

The JNDs in the time-to-arrival discrimination task indicated that participants could on average discriminate time-to-arrival differences of 0.72 s (*SD* = 0.31 s), with a good fit of the psychometric functions to the data of all participants (mean *SE* = 0.11 s, min = 0.05, max = 0.40; mean *Dev* = 7.78, min = 1.94, max = 13.12, all *p* > 0.050). The corresponding average Weber fractions (see Fig.  were 0.38 (95% CI [0.25, 0.51]) for speed discrimination and 0.24 (95% CI [0.18, 0.29]) for time-to-arrival discrimination, respectively (see Fig. 3C). A Wilcoxon signed rank sum test indicated that Weber fractions for the majority of participants were higher for speed discrimination compared to time-to-arrival discrimination (*z* = 2.45, *p* < 0.010, *n* = 14).

As noted above, the standard and comparison were identical except for the differences in the vehicle speed and time-to-arrival and therefore we did not expect any response bias for the comparison interval when the time-to-arrival or speed were the same as the standard, i.e., we expected the PSE to be at the standard level. The confidence interval for the PSE in speed (*mean* = 49.27 km/h, 95% CI [47.06, 51.49 km/h]) and time-to-arrival judgements (*mean* = 3.08 s, 95% CI [3.02, 3.14 s]) contained the standard speed (50 km/h) and time-to-arrival (3.04 s) confirming that there was no response bias.

We further investigated the presence of an interval bias by fitting the psychometric functions to the proportion of times the vehicle in the *second* interval was judged to move faster or to arrive earlier. The confidence interval for the PSE in the speed task (*mean* = 46.14 km/h, 95% CI [43.87, 48.40 km/h]) indicated a slight tendency to select the second interval independently of the difference in the speed between comparison and standard interval. The confidence interval in the time-to-arrival task (*mean* = 3.71 s, 95% CI [3.15, 4.26 s]) indicated that participants had as well a tendency to respond that the vehicle in the second interval would have arrived earlier.

### Influence of motion and position cues on speed and time-to-arrival judgements

By combining the nine speed and time-to-arrival levels, the approach speed of the comparison vehicle and its remaining time-to-arrival after the fixed display interval varied independently. Due to the nature of this manipulation, however, the vehicle’s start and end position could not be independent of its speed and time-to-arrival (see Fig. 2), which rendered the vehicle position a cue for the vehicle’s speed and time-to-arrival, albeit not always a reliable one. We explored how participants took these parameters into account by performing logistic regressions entering vehicle speed, time-to-arrival as well as start and end position as variables for predicting each participant's “faster” (speed discrimination task) and “earlier” (time-to-arrival discrimination task) responses. Subsequently, we performed dominance analyses to quantify the relative contribution of the different factors to the model fit. For comparison, we performed the same analyses for a theoretical observer who would respond “faster” (or “earlier”) whenever the comparison vehicle was faster (or earlier) than the standard vehicle. To this observer model, we added a source of Gaussian noise with a mean of 0 and a standard deviation that varied per participant and was estimated from the standard deviation of the Gaussian fit to the actual proportion “faster” or “earlier” responses. This allowed us to evaluate the importance of each cue relative to a theoretical observer that would only respond based on the instructed cue (vehicle speed or time-to-arrival), meaning that any relevance of other cues in the theoretical observer would only reflect co-variation between cues and not the participant’s strategy.

**Figure 4:**
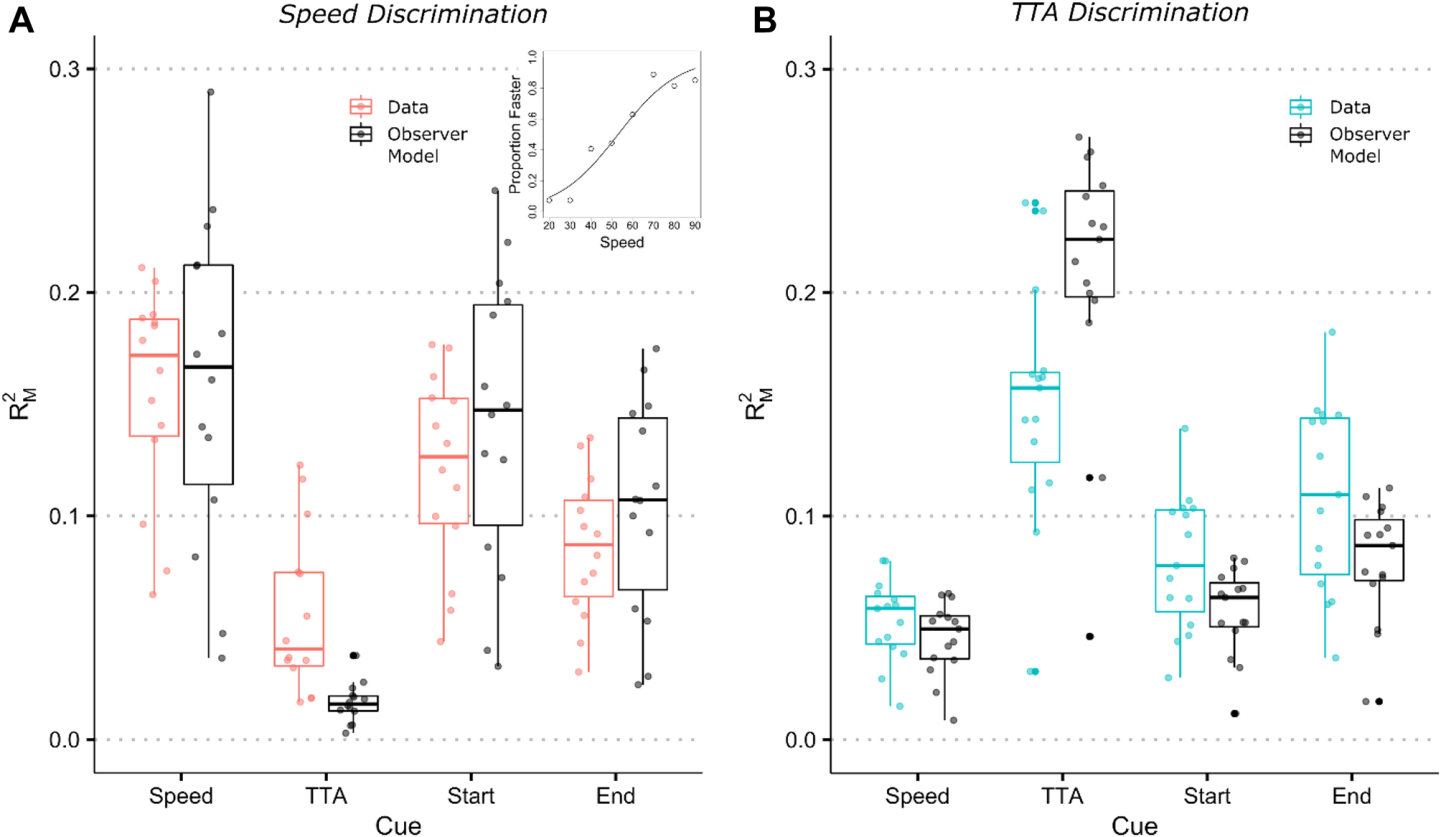
Average contribution of vehicle speed, time-to-arrival, start and end distance of the comparison vehicle to the model fits (R^2^_M_) predicting the probability of “faster” or “earlier” responses. Average contributions are plotted as dots for each participant in (**A**) the speed discrimination task (*N* = 14) and (**B**) the time-to-arrival discrimination task (*N* = 15). Crossbars indicate the median. Whiskers depict the 1.5﻿ ×﻿ interquartile ranges. For comparison, average contributions are also plotted for a theoretical observer model responding only to the vehicle speed or time-to-arrival. The inserted panel depicts a general linear model fit for one exemplar participant predicting the proportion “faster” responses as a function of vehicle speed.

Figure 4 plots the general dominance measures, i.e., the average conditional contribution of each predictor to all subset model fits based on the R^2^_M_ index (an R^2^ analogue for logistic regression), for both the participants and theoretical observer models^41^. For speed judgements, we found that vehicle speed was the generally dominant predictor for participants’ responses (*mean* = 0.16, 95% CI [0.13, 0.18]) followed by the start (*mean* = 0.12, 95% CI [0.10, 0.15]) and end distance of the vehicle (*mean* = 0.09, 95% CI [0.07, 0.10]). Although vehicle speed and time-to-arrival varied independently, participants based their judgements more on the time-to-arrival of the vehicles (*mean* = 0.06, 95% CI [0.04, 0.08]) than would have been expected from a theoretical observer that only responded to vehicle speed (*mean* = 0.02, 95% CI [0.01, 0.02]). In terms of time-to-arrival judgements, we found that participants responses were considerably less well predicted by the actual time-to-arrival of the vehicle (*mean* = 0.15, 95% CI [0.12, 0.18]) compared to a theoretical observer responding based on time-to-arrival alone (*mean* = 0.21, 95% CI [0.18, 0.24]). Instead, the use of vehicle position, i.e., start (*mean* = 0.08, 95% CI [0.06, 0.10]) and end distance (*mean* = 0.11, 95% CI [0.09, 0.13]) of the vehicle, was more dominant than expected from the respective observer models (start: *mean* = 0.06, 95% CI [0.05, 0.07]; end: *mean* = 0.08, 95% CI [0.07, 0.09]). Vehicle speed affected time-to-arrival judgements only little (*mean* = 0.05, 95% CI [0.04, 0.06]).

### Comparison of eye movements during perceptual judgements

**Figure 5:**
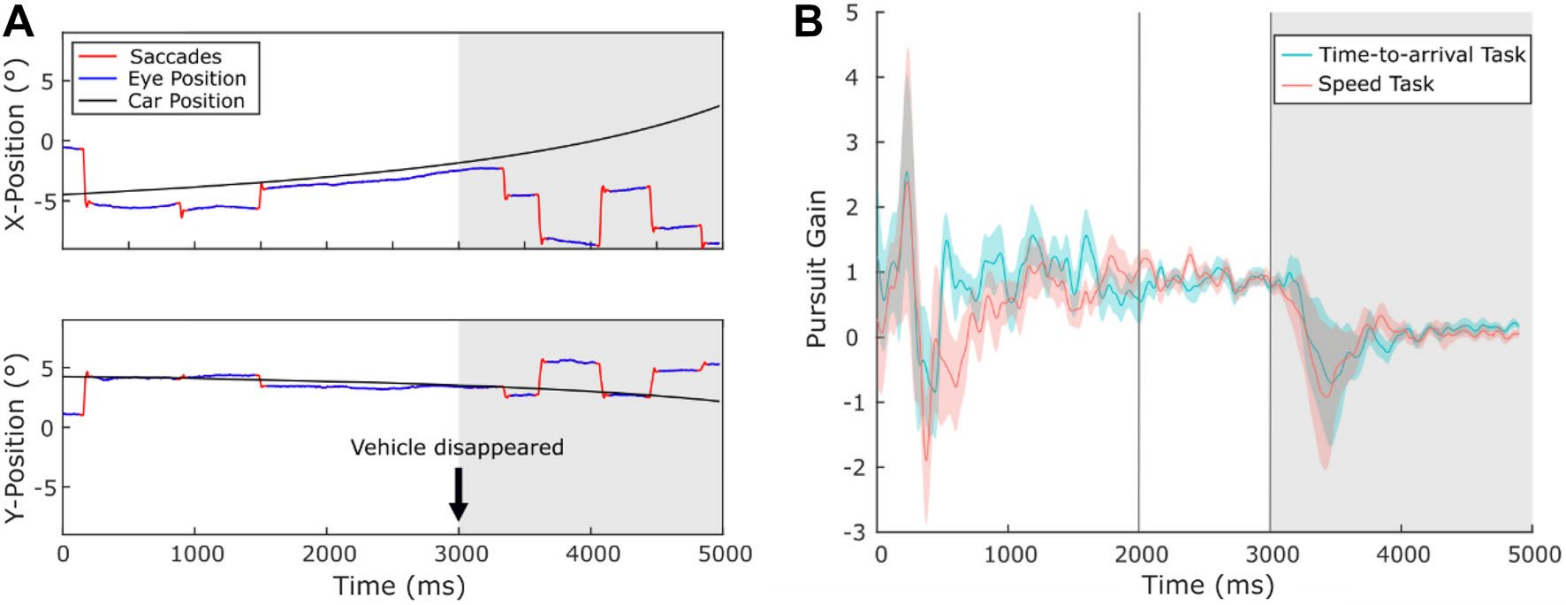
Example of eye movement traces and average pursuit gain. (**A**) Shows the horizontal and vertical vehicle trajectory and unprocessed eye movement traces of one participant in degrees of visual angle. The solid black line denotes the visible vehicle trajectory. The dotted black line denotes the extrapolated vehicle trajectory (assuming constant velocity) after the vehicle disappeared. Saccades are marked in red. (**B**) Shows pursuit gain averaged over participants (*N* = 14) and trials. Shaded areas indicate standard errors of the sample means. The 2–3 s time interval before the car disappeared was used as the averaging interval for pursuit gain, relative and absolute position deviations.

Figure 5A shows the eye movement traces of one exemplar participant. Low absolute and relative position deviations indicate that in both conditions participants fixated closely towards the vehicle’s AOI center. As the approaching vehicle came closer, participants tracked it via a combination of smooth pursuit and catch-up saccades. Figure 5B shows pursuit gain averaged over all participants and trials during the speed and time-to-arrival discrimination task. After an initial deflection, which is likely artefactual and resulting from dividing the gaze velocity during the orientation towards the vehicle by a very slow retinal velocity of the vehicle approaching from a distance, pursuit gain increased to an average value of around 0.9 in both conditions. After 3 s the vehicle disappeared and participants gain decayed. An overshoot in the gain decay can be observed when the stopping of the pursuit target is unpredictable^48^. Although in our study all vehicles disappeared after a fixed display time of 3 s, the travelled distances of the vehicles varied and the overshoot in the gain decay is therefore likely to be explained by the unpredictability of the disappearance event. In both conditions, saccades towards the road crossing AOI were performed only in few trials (3% of all trials) and occurred mostly after the approaching vehicle disappeared.

Descriptive statistics of the averaged eye movement data showed little differences in participants’ gaze behavior between speed and time-to-arrival judgements. To compare the eye movement measures between conditions while accounting for multiple comparison testing, we performed a repeated measures MANOVA. Using Wilks’s Lambda, we found no overall significant effect of task on eye movement behavior (Ʌ = 0.362, *F*(9,5) = 0.98, *p* = 0.541). All univariate comparisons of gaze measures were also non-significant (all *p* > 0.05, see Table 1 for descriptive statistics and F-values).

**Table 1:** Means, standard deviations and F-statistics from univariate comparisons of eye movement measures.

	Speed		Time-to-arrival		*F*(1,13)	*p*
	*M*	*SD*	*M*	*SD*		
Gain	0.90	0.12	0.84	0.13	2.54	0.135
**Rel. position deviation (°)**						
Vertical	0.68	0.53	0.72	0.53	0.09	0.767
Horizontal	0.53	0.11	0.51	0.16	0.02	0.900
**Abs. position deviation (°)**						
Vertical	0.95	0.30	0.96	0.37	0.02	0.906
Horizontal	1.35	0.45	1.44	0.42	0.82	0.383
Number of saccades	2.96	1.01	3.02	1.20	0.24	0.631
Amplitude of saccades (°)	1.09	0.28	1.13	0.23	1.11	0.311
**Number of saccades to road-crossing area**						
Before car offset	0.01	0.01	0.01	0.01	< 0.01	0.975
After car offset	0.04	0.06	0.04	0.06	< 0.01	0.969

### Predicting the observer and perceptual task from eye movements

**Figure 6:**
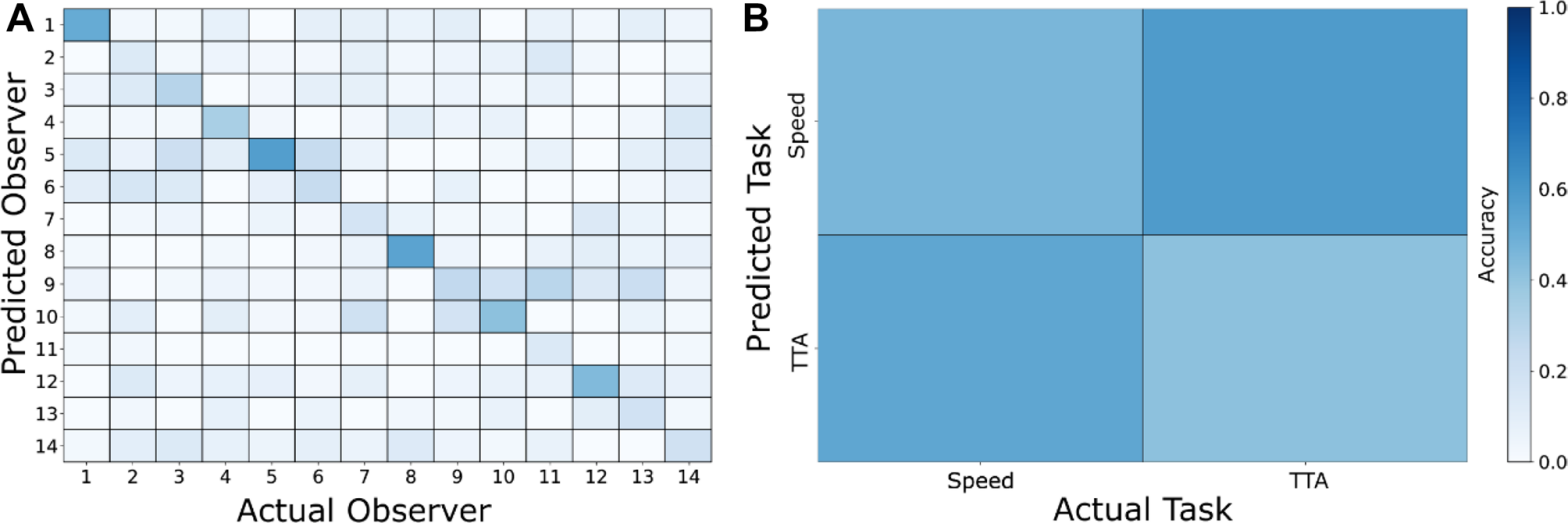
Confusion matrices of SVM predictions. (**A**) Shows the observer classification performance. (**B**) Shows the task classification performance.

Variability between observers in their individual gaze patterns may impede the detection of overarching differences between the two perceptual tasks. To explore the prevalence of idiosyncratic gaze patterns, we trained an SVM to predict the observer based on the eye movement features listed in Table 1. We trained the classifier to perform a non-linear classification using a radial kernel and tested its performance using a tenfold cross validation procedure. The SVM was able to determine the observer significantly above the level that would have been expected without prior training (Accuracy = 0.31, CI [0.30, 0.33], No Information Rate = 0.09, *p*(one-sided) < 0.001). As indicated by the permutation test, the classifier was also significantly better at predicting the observer than it would have been expected under the null hypotheses assuming that eye movements and observers were independent (*p*(one-sided) < 0.001). As shown in Fig. 6A, idiosyncratic gaze patterns were distinct enough to identify individual observers.

We trained a second classifier to predict the perceptual task using the same eye movements features and model specifications. Only in 2 out of 14 participants we obtained predictions that exceeded the no information rate, suggesting that differences in the eye movement recordings were not sufficient in the majority of participants to discriminate between the two tasks. The average accuracy of predictions across participants was even below the no information rate (Accuracy = 0.48, 95% CI [0.45 0.49], No Information Rate = 0.52; see Fig. 6B) and thus, there was no need to test whether the classifier performed significantly better. The permutation test indicated that the average prediction accuracy did not exceed the accuracy expected for independent eye movement and task labels (*p*(one-sided) = 0.500).

Both algorithms used a relatively high percentage of observations as support vectors (observer classification: 69%, task classification: 59%), indicating that in both cases a high number of training samples was necessary to build the classification models.

### Influence of eye movements on performance

To investigate the influence of gaze behavior on discrimination performance, we specified two logistic regression models predicting the likelihood of correct responses in each task from eye movement measures. We excluded the number of saccades towards the road crossing AOI as these occurred only very infrequently. Using a generalized linear mixed model (GLMM) approach, we added random effects for the observer as well as the speed and time-to-arrival of the comparison vehicle to the models to ensure the generalization of the results to different observers, vehicle speeds and arrival times. Our final models included the number and amplitude of saccades, relative and absolute horizontal and vertical position deviations as well as pursuit gain as fixed effects and observer, vehicle speed and time-to-arrival as random effects. All included fixed effects measures were averaged across the interval in which the vehicle was visible.

The maximal model did not converge for neither the speed nor time-to-arrival task. A common and often recommended first step is to simplify the random effects structure by dropping the assumed correlation between intercepts and slopes, i.e., constraining the correlation parameters between the intercepts of random factors and coefficient estimates to zero^49^. With the reduced models we achieved convergence. A comparison between the models and more parsimonious models with a further simplified random effects structure indicated that a further reduction did not improve the model fits, as demonstrated by similar or lower likelihoods and similar or higher Akaike information criteria (AICs) for the reduced models, which underpinned our final model selection. Multicollinearity among the fixed predictors was measured by the variance inflation factors (VIF), which provided no substantial evidence of multicollinearity in the two models (speed model: all VIF < 1.21; time-to-arrival model: all VIF < 1.81).

**Figure 7:**
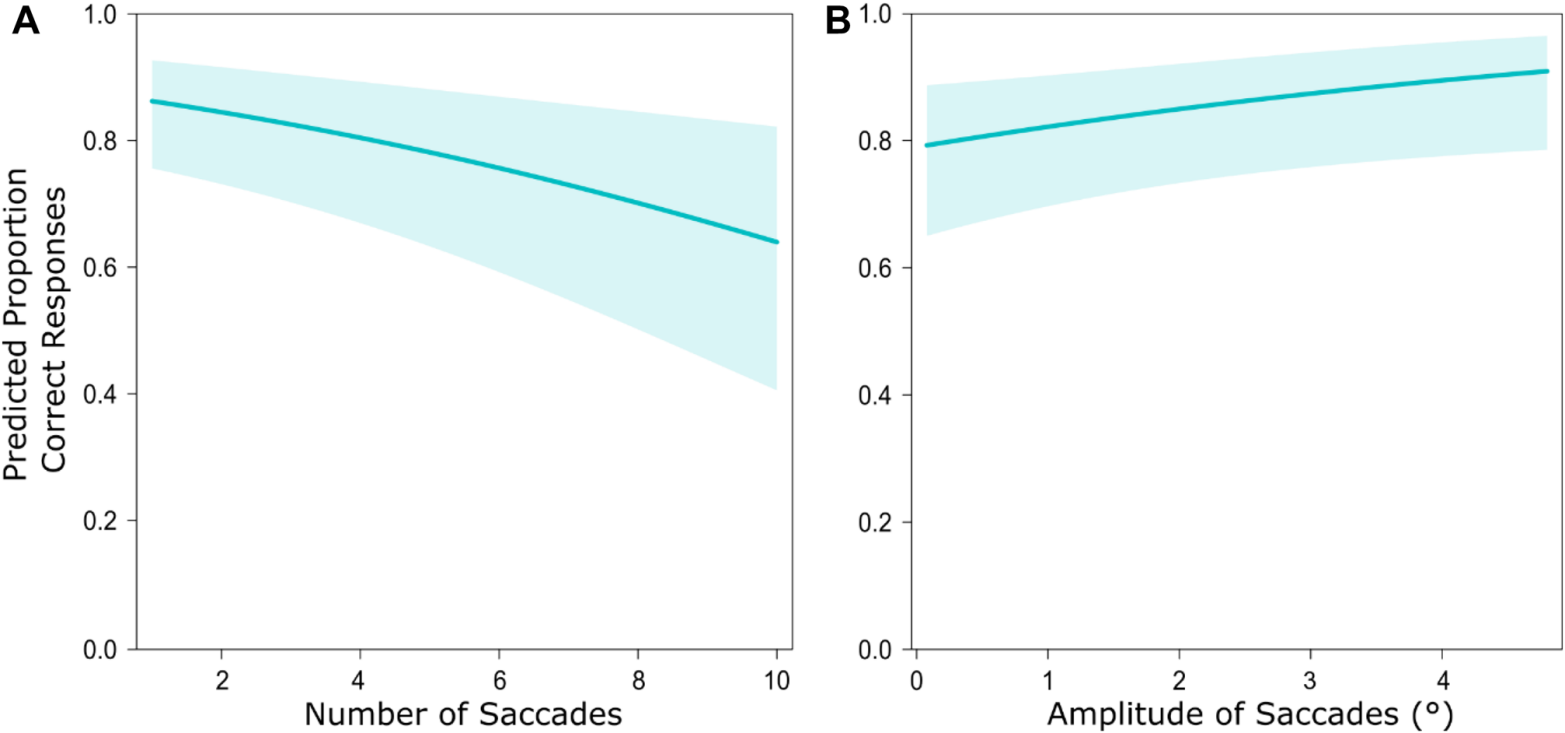
Effects of gaze behavior towards the comparison vehicle on discrimination performance. Panels depict the predicted proportion of correct responses in the time-to-arrival discrimination task by (**A**) the number saccades and (**B**) the amplitude of saccades. Shaded areas represent the 95% confidence interval.

All parameter estimates from our final models are reported in Table 2. In the time-to-arrival model, the number and amplitude of saccades were significant predictors for discrimination performance suggesting that performance increased with a decreasing number of saccades and with increasing amplitudes (see Fig. 7). In the speed task, none of the included eye movement measures could predict correct responses.

**Table 2:** Effects of eye movements during the comparison interval on correct discrimination (GLMM analysis).

	Model speed				Model time-to-arrival			
	*Estimate*	*SE*	*z*	*p*	*Estimate*	*SE*	*z*	*p*
(Intercept)	1.47	0.34	4.31	< 0.001***	1.79	0.38	4.62	< 0.001***
Gain	− 0.04	0.06	− 0.67	0.506	0.07	0.06	1.14	0.254
Number of saccades	0.05	0.04	1.07	0.283	− 0.14	0.05	− 3.13	0.002**
Amplitude of accades (°)	− 0.10	0.09	− 1.17	0.244	0.21	0.10	2.08	0.037*
**Rel. position deviation (°)**								
Horizontal	− 0.06	0.11	− 0.59	0.558	− 0.04	0.07	− 0.48	0.633
Vertical	0.09	0.17	0.55	0.581	− 0.13	0.13	− 0.95	0.342
**Abs. position deviation (°)**								
Horizontal	0.02	0.15	0.13	0.896	− 0.01	0.07	− 0.14	0.889
Vertical	1.51	0.19	0.82	0.411	0.02	0.15	0.14	0.886

*Significant at *p* < 0.05. **Significant at *p* < 0.01. ***Significant at *p* < 0.001.

## Discussion

The present study addressed the question of how pedestrians sample visual information about the speed and time-to-arrival of approaching vehicles and how eye movements influence perceptual judgements in road traffic. We tested speed and time-to-arrival discrimination of approaching vehicles as seen from the side of a road and explored the spontaneous gaze behavior that subtends those judgements.

### Discrimination and cue use

Assessing the discrimination thresholds (JNDs) for speed and time-to-arrival judgements, we found that observers were able to discriminate vehicle speed differences of 18.08 km/h and time-to-arrival differences of 0.74 s. The corresponding Weber fractions were larger for speed discrimination (38%) than for time-to-arrival discrimination (24%) and considerably exceeded the speed discrimination sensitivities reported previously for abstract moving stimuli (5–12%)^50–52^. We assume that the simulated perspective in our road-crossing scenario may have contributed to the comparably low speed discrimination sensitivities. When standing at the edge of a road, pedestrians typically view vehicles approaching from an oblique angle. Compared to translational motion presented on the frontoparallel plane, the physical speed does not directly translate to retinal image speed and retinal speeds are less indicative of physical speed the farther away the vehicles are from the observer. Previous research investigating speed discrimination of looming stimuli demonstrated that observers draw on sub-optimal strategies in such cases, e.g., relying on the distance or size of a moving object^53^. Although we observed that vehicle speed was the main predictor for speed judgements, responses were also biased by the time-to-arrival of the vehicles, indicating that observers drew on other, irrelevant cues for speed discrimination. The lack of stereoscopic depth cues and relative scarcity of our virtual scenario may have further hampered factoring out distance when comparing vehicle speeds^54^. In a real-life road-crossing scenario we may expect discrimination to improve when further distance and depth cues are available. Moreover, the increased visual angle the vehicles would span when viewed in real-life would provide a stronger motion signal than in our setup. For example, at a simulated distance of 50 m from the observer, the image of the vehicle’s front plane spanned around 0.95 degrees of visual angle (as viewed from a 66 cm distance from the display). When transferred to real-life, the vehicle’s front plane would span around 2 visual degrees. Although the differences in visual angles covered by the front plane of the vehicle were negligible when vehicles were far away, they increased as the vehicle approached the observer. It remains to be tested whether a more realistic simulation of the viewing angles at vehicle distances relevant for road-crossing decisions would improve speed discrimination.

In terms of time-to-arrival discrimination, we found that responses were considerably less well predicted by the actual time-to-arrival of the vehicles than expected from an unbiased response model, i.e., a theoretical observer responding to the time-to-arrival alone. This was to the benefit of position cues, i.e., the start and end distance of the vehicles to the stopping line, which were on average more predictive of responses than expected by their covariation with time-to-arrival alone, indicating that observers overly relied on vehicle distance. An overuse of distance information in time-to-arrival judgements has been previously demonstrated for both abstract motion stimuli^55^ and simulated vehicles even if other sensory cues, such as auditory information, are present^56,57^. However, a comparably high variation across observers in terms of the predictor importance of the start and end distance of the vehicles compared to the theoretical observer model suggested that observers differed in their use of positional cues. This is in line with previous studies reporting on the flexibility of strategies used for estimating time-to-arrival^58^ and suggests that perceptual strategies employed to evaluate whether the approaching traffic leaves sufficient time to cross may not necessarily be universal among pedestrians even under similar conditions.

For both types of judgements, we observed a small temporal-order effect indicating that the comparison vehicle was more likely judged to drive faster or to arrive earlier than the standard when displayed on the second interval. This bias is consistent with previous research showing that observers tend to select the second interval in similar types of judgements^59^ and may reflect the indecisiveness of our observers in their perceptual judgements^39^.

### Comparison of eye movements during perceptual judgements

We tested whether different perceptual judgements would result in distinctive gaze patterns and whether the motion parameter an observer evaluates could be predicted from eye movements. We observed little differences in the averaged eye movement measures between speed and time-to-arrival judgements demonstrating that visual sampling was similar during both perceptual tasks. Instead, the individual gaze patterns of participants were more pronounced than the differences in the eye movement behavior between the two tasks. Across tasks, the SVM classifier could identify observers with an accuracy of 31%, which was well above chance level. Although in our simulated scenes visual features were relatively scarce, this finding suggests that the stimuli still allowed for sufficient inter-individual variability to discriminate gaze behavior between participants. In contrast, the accuracy of task classification was only 48% and not better than chance. The low task classifier performance is in line with our comparison of the averaged eye movement data and suggests that there were no overarching differences in the assessed gaze behavior that could discriminate between the perceptual tasks across participants.

During both tasks, participants commonly fixated closely towards the center of the vehicle’s front plane and tracked it via a combination of smooth pursuit eye movements and catch-up saccades when it came closer. Prior studies exploring eye movements during time-to-arrival and motion prediction tasks reported saccades towards the crossing point as a characteristic feature of gaze behavior^11,15^. We did not observe such a behavior in our participants. Saccades towards the crossing area occurred very infrequently and could be observed only on a very low number of trials independent of the performed perceptual task. On the one hand, visual attention towards the crossing area might have played a minor role for time-to-arrival judgements in our study since observers were asked to perform relative judgements instead of predicting absolute arrival times. In that sense, the visual information provided by the vehicles, such as their relative size, looming and image speed, could have been sufficient to solve the discrimination task. On the other hand, foveating the vehicles did not necessarily exclude that observers simultaneously assessed the location of the crossing point. Instead, due to the relative simplicity of our traffic scenarios, the location of the crossing point may have already been sufficiently available from peripheral vision^60–62^. While a more detailed sampling of the crossing point may aid motion prediction when observers respond to the arrival of a target^11^ or when self-motion during driving needs to be taken into account^15^, foveating the approaching vehicles while the crossing point was peripherally available was the preferred sampling strategy for relative arrival time judgements in our study and gaze behavior therefore differed little from the sampling strategy performed during speed judgements.

### Influence of eye movements on performance

The association of perceptual judgements with eye movement metrics, such as pursuit gain, number and amplitude of saccades and gaze location, have been well-documented both in fundamental (with abstract stimuli) and applied contexts (with naturalistic stimuli). We explored how these relations generalize to pedestrians in a simple road-crossing scenario by modelling the effects of eye movements on the likelihood of correct discriminations. Smooth pursuit is suggested to aid motion prediction by providing an extra-retinal motion signal in addition to retinal motion^10^ and improved performance during smooth pursuit compared to fixation has been previously demonstrated for time-to-arrival estimations^8,9^. When modeling the effects of eye movements, we did not observe an effect of pursuit gain on discrimination performance neither in the speed nor the time-to-arrival task, indicating that matching the velocity of gaze to the vehicle velocity did not noticeably improve performance. Our stimuli were designed to resemble the view of a pedestrian standing close to the road and the retinal velocity of the simulated vehicles in our study was at times very low due to the simulated approach angle, ranging between 0.07 and 1.22 deg/s averaged across the first second of display and resulting in an almost static retinal image when vehicles were far away. We conclude that in a comparable road-crossing scenario, smooth pursuit may therefore play a minor role for motion discrimination unlike it does with the higher retinal velocities commonly used in pursuit studies.

Low positional deviations between gaze and vehicle position indicated that participants kept the vehicle foveated throughout approach and placed their gaze close the center of the vehicle’s front plane. This is in line with previous research demonstrating that observers naturally fixate towards the front plane of comparably sized vehicles when discriminating vehicle speeds^16^. We show that gaze was spontaneously directed towards the same position when observers discriminated the times-to-arrival of vehicles. Depending on the gaze location, the image speed of a moving object on the retina differs. Previous studies showed that gaze positions differ between small and large vehicles, which may explain perceptual biases experienced in traffic such as the size-speed illusion^16,17^. In terms of discrimination between same-sized vehicles, however, our models indicated no effects of gaze position on performance. One explanation could be that the low variability of participants’ gaze position made it difficult to detect any positional effects. On the other hand, and especially for time-to-arrival judgements, it might have also been that the positional and looming information of the vehicle played a more important role for discrimination than retinal image speed, as indicated by the correlation of judgements with visual cues.

The number and amplitudes of saccades had a significant effect on performance in time-to-arrival discrimination. Participants were more likely to respond correctly when they performed fewer saccades and when the amplitudes of saccades were larger. Previous studies demonstrated that saccade execution is associated with a compression of the perceived time and distance between visual stimuli^12,13^, suggesting that saccades may as well impair accurate time-to-arrival judgements. Simultaneously, the compression varies only little with the amplitude of saccades. A successful gaze strategy to estimate time-to-arrival in our scenario may have therefore been to fixate towards the vehicle and update gaze position with fewer, but larger amplitude saccades as the vehicle approached. As the retinal speeds were small, this would have allowed participants to keep the vehicle foveated while minimizing perceptual distortions during saccades.

We assumed that gaze behavior during the standard interval would be less indicative of performance due to observers being able to internalize the motion of the standard vehicle during the course of the experiments. We therefore primarily focused on the analyses of gaze data from the comparison interval. Visual sampling during the standard interval was overall similar to the comparison interval (see supplementary material) and as well failed to accurately discriminate between tasks. Performance in the time-to-arrival task could not be predicted from gaze behavior during the standard interval. In the speed discrimination task, the horizontal deviation of gaze from the vehicle and the number of saccades had a statistically significant effect on performance, suggesting that keeping the standard vehicle foveated and tracking the center of its front plane improved speed discrimination performance.

### Limitations and transferability of the results to real road environments

Our results can give an indication as to how pedestrians sample visual information when evaluating the speed and time-to-arrival of approaching traffic and on the discriminability of these motion parameters when assessed from a pedestrian’s perspective. The question remains of how transferable the results are to an actual road environment. The simulated scenes used in our experiment were somewhat idealized, in the sense that they provided a clear visibility of the vehicle, which approached with a constant driving speed on a straight, single-lane road. Apart from basic textures and road markings, other visual elements in the scene were scarce, with no road signs, buildings or vegetation. Stimuli were displayed on a 2D monitor and hence, no binocular depth cues were available and viewing angles were smaller compared to a comparable real-life scenario. In terms of sensitivity, some of these factors may have impaired discrimination (e.g., the lack of binocular depth cues and visual elements in the road environment providing further distance information, smaller viewing angles), while others may have enhanced it (e.g., the absence of visual crowding and attentional competition). Further studies are needed to compare our discrimination thresholds to the sensitivities encountered in a real road-environment. With regard to previous research and our own findings, we would expect different road environments to yield considerably different sensitivities depending on the availability of visual cues in the specific scenario, which may as well interact with the individual cue use and gaze behavior of the observer.

In terms of gaze behavior, we found that eye movements could not be used to discriminate between perceptual judgements. We selected a discrimination task for this comparison as it enabled us to present participants with a similar task procedure that only differed in respect of the motion parameter in question. But how relevant is motion discrimination for road-crossing decisions? We suppose that a discrimination task resembles the informational requirements for identifying appropriate gaps between consecutive vehicles, which is suggested to incorporate an estimate of the difference between the time-to-arrival of a leading and following vehicle^63^. Gap selection would thus require a relative rather than absolute estimate of time-to-arrival to identify the most favorable opportunity for crossing. On the one hand, an absolute estimate of each vehicle’s arrival time might be a better representation of the perceptual requirements for collision avoidance. The sampling strategies we observed during relative time-to-arrival judgements differed from the gaze behavior reported earlier on absolute time-to-arrival judgements in motion prediction tasks^11^. If it was the difference in the perceptual requirements of the tasks that elicited the different sampling strategies, eye tracking could bring some clarification as to what role absolute and relative judgements play for crossing-decisions. On the other hand, we cannot rule out that the similarity of the sampling strategy employed for time-to-arrival judgements with speed judgements resulted from the simplicity of our scenario. It might be that eye movements would have been more distinct if the crossing area was less well represented in the periphery, e.g., due to a higher eccentricity or a higher degree of visual crowding^60,61^. Further studies are needed to clarify whether the demands of the task or the virtual scene promoted the observed sampling behavior in which overt spatial attention was almost exclusively directed towards the approaching vehicles. With an increased field of vision and a more complex road scenario we may also expect not only eye movements but also head movements to be performed for visual sampling. When transferred to real-life, the angular size of the visible trajectories as displayed in our road scenario are below a visual angle for which we would expect a considerable contribution of head rotations for foveating the approaching vehicles (~ 20 visual degrees)^64^. Nevertheless, head rotations could play a role in a more complex crossing task, e.g., for monitoring traffic from multiple directions and for preparing and guiding self-motion. To generalize to a more complex road-crossing scenario, future studies should therefore take not only eye movements but also head movements into account when assessing the effects of visual sampling behavior on perceptual judgements and road-crossing decisions of pedestrians.

## Conclusion

When viewing a simulated traffic scene from a pedestrian’s point of view, observers could discriminate differences in vehicle speed of around 18 km/h and time-to-arrival differences of around 0.7 s. Compared to what has been previously reported with abstract motion stimuli, observers discriminated relatively poorly between vehicle speeds in our simulated scenario and responses were biased by irrelevant cues, such as the vehicle’s time-to-arrival. With regard of time-to-arrival judgements, observers varied in respect to their use of positional information and idiosyncrasies in spontaneous eye movements were sufficient to distinguish between observers. We found no differences in visual sampling that were sufficient to discriminate between the two tasks. Both the speed and time-to-arrival of vehicles were similarly sampled by keeping the gaze close to the center of the vehicle’s front plane. While pursuit and gaze position played a minor role for motion discrimination in the simulated road-crossing scenario, saccadic gaze behavior could predict time-to-arrival discrimination performance. A successful visual sampling strategy involved few, but larger amplitude saccades. We conclude that understanding how observers sample and integrate visual information about approaching vehicles can provide valuable insights into the perceptual bases for navigation and collision avoidance of pedestrians in traffic. Our study  shows to which extentbasic vision research and driver simulation studies can predict pedestrians' perception and eye movement behaviour in a naturalistic but very simple traffic scenario. Further studies are needed to explore visual sampling and perceptual judgments in more complex road-crossing scenarios.

## Supplementary Information

Supplementary Information 1.
