# Supplementary material for: The role of eye movements in perceiving vehicle speed and time-to-arrival at the roadside
Source: Sci Rep. 2021 Dec 2;11:23312. doi: 10.1038/s41598-021-02412-x (PMC8640052; doi:10.1038/s41598-021-02412-x)
Supplement: Supplementary file 1 — Supplementary Information 1. [file 41598_2021_2412_MOESM1_ESM.docx]

**The role of eye movements in perceiving vehicle speed and time-to-arrival at the roadside**

Jennifer Sudkamp, Mateusz Bocian, David Souto

# SUPPLEMENTARY MATERIAL

## Gaze analyses on eye movement data recorded during the standard interval

### Data Preparation

We used the same criteria for outlier detection as for the comparison interval. On average, 3.06% (min = 1.68%, max = 5.23%) of data points per observer were removed for the standard interval data.

### Comparison of eye movements between perceptual judgements

The discrimination task had no significant effect on gaze behavior during the standard interval (repeated measures MANOVA: Ʌ = .714, *F*(9,5) = 1.39, *p* = .376). All univariate comparisons of gaze measures between perceptual judgements, except for the horizontal relative position deviation (*F*(1,13) = 4.83, *p* = .047), were also non-significant (all other *p* > .05, see Table S1 for means and F-statistics). Figure S1 depicts pursuit gain during the standard interval averaged over participants and trials.

Table S1.

Means, standard deviations and F-statistics from univariate comparisons of eye movement measures between perceptual judgements.

|  | Speed | | Time-to-arrival | |  | |  |
| --- | --- | --- | --- | --- | --- | --- | --- |
|  | *M* | *SD* | *M* | *SD* | *F*(1,13) | *p* | |
| Gain | 0.90 | 0.12 | 0.94 | 0.17 | 0.99 | .338 | |

| Rel. position deviation (°) |  |  |  |  |  |  |
| --- | --- | --- | --- | --- | --- | --- |
| Vertical | 0.38 | 0.54 | 0.43 | 0.50 | 0.22 | .645 |
| Horizontal | -0.07 | 0.06 | 0.01 | 0.14 | 4.83 | .047* |
| Abs. position deviation (°) |  |  |  |  |  |  |
| Vertical | 0.68 | 0.29 | 0.70 | 0.31 | 0.06 | .812 |
| Horizontal | 0.80 | 0.59 | 0.94 | 0.56 | 0.88 | .365 |
| Number of saccades | 2.78 | 1.01 | 2.97 | 1.21 | 2.84 | .116 |
| Amplitude of saccades (°) | 0.93 | 0.26 | 1.00 | 0.23 | 4.30 | .059 |
| Number of saccades to road crossing area |  |  |  |  |  |  |
| Before car offset | <0.01 | <0.01 | <0.01 | 0.01 | 0.72 | .411 |
| After car offset | 0.05 | 0.07 | 0.04 | 0.05 | 0.43 | .524 |

*Note*. * Significant at *p* < .05.


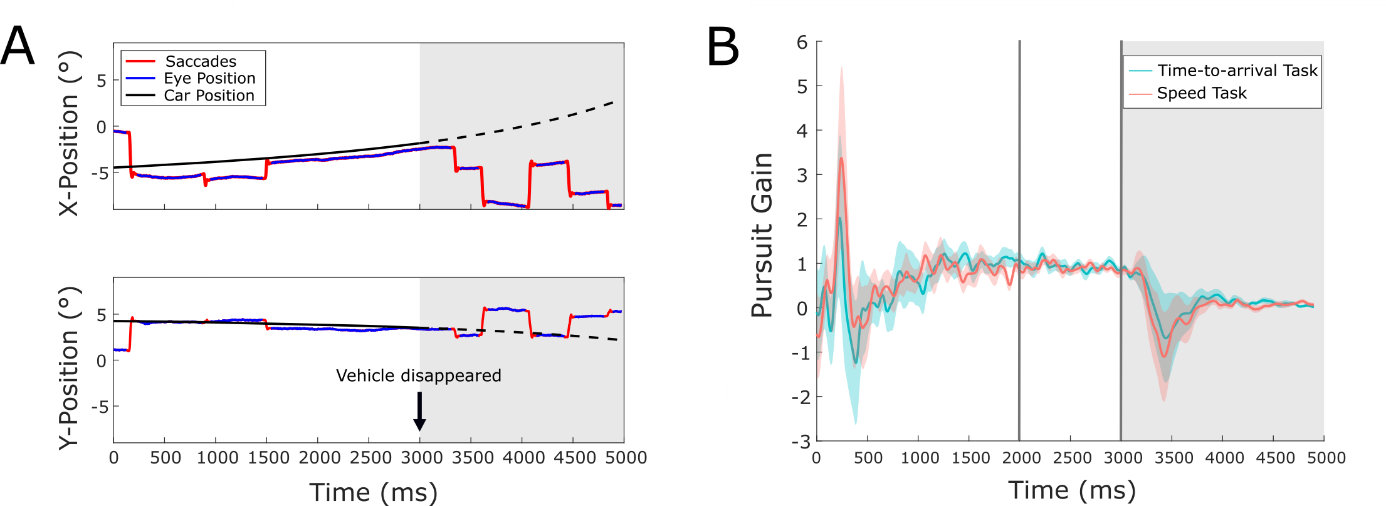


**Figure S1.** **Average pursuit gain during the standard interval.** Pursuit gain averaged over all participants (*N* = 14) and trials. Shaded areas indicate standard errors of the sample means.

### Predicting perceptual task from eye movements

Observer classification of the trained SVM significantly exceeded the no information rate (Accuracy = .42, CI [.41, .43], No Information Rate = 0.09, *p*(one-sided) < .001). The permutation test showed that the classifier was also significantly better at predicting the observer than it would have been expected under the null hypotheses assuming that eye movements and observers were independent (*p*(one-sided) < .001).

In terms of task classification, the average accuracy of predictions was only slightly higher compared to the no information rate (Accuracy = .53, 95% CI [.51, .56], No Information Rate = .52, *p*(one-sided) = .290). The permutation test nevertheless indicated that the average prediction accuracy exceeded the accuracy expected for independent eye movements and task labels (*p*(one-sided) = .001). Figure S2 shows the confusion matrices for the trained SVMs.


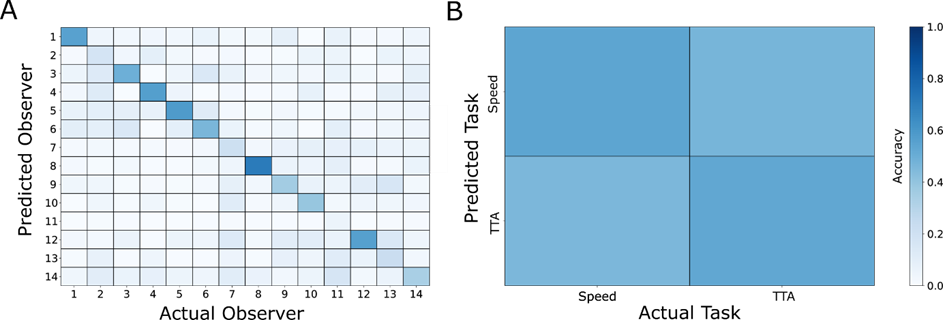


**Figure S2. Confusion matrices of SVM predictions.** (A) shows the observer classification performance. (B) shows the task classification performance.

### Influence of eye movements on performance

Models included the number and amplitude of saccades, relative and absolute horizontal and vertical position deviations as well as pursuit gain as fixed effects and observer as random effects. All included fixed effects measures were averaged across the interval in which the vehicle was visible. All parameter estimates are reported in Table S2.

Table S2.
*Effects of eye movements during the standard interval on correct discrimination (GLMM analysis).*

|  | **Model Speed** | | | |  | **Model Time-to-Arrival** | | | |
| --- | --- | --- | --- | --- | --- | --- | --- | --- | --- |
|  | *Estimate* | *SE* | *z* | *p* |  | *Estimate* | *SE* | *z* | *p* |
| (Intercept) | 0.87 | 0.18 | 4.71 | <.001*** |  | 1.38 | 0.15 | 9.41 | <.001*** |
| Gain | -0.01 | 0.04 | -0.14 | .887 |  | -0.00 | 0.04 | -0.00 | .997 |
| Number of saccades | 0.09 | 0.04 | 2.38 | .017* |  | 0.01 | 0.03 | 0.15 | .883 |
| Amplitude of saccades (°) | 0.19 | 0.10 | 1.95 | .052 |  | -0.06 | 0.09 | -0.63 | .529 |
| Rel. position deviation (°) |  |  |  |  |  |  |  |  |  |
| Horizontal | -0.02 | 0.04 | -0.53 | .594 |  | 0.01 | 0.04 | 0.36 | .720 |
| Vertical | 0.20 | 0.13 | 1.55 | .120 |  | -0.01 | 0.10 | 0.13 | .900 |
| Abs. position deviation (°) |  |  |  |  |  |  |  |  |  |
| Horizontal | -0.22 | 0.09 | -2.49 | .013* |  | -0.12 | 0.06 | -1.92 | .056 |
| Vertical | 0.06 | 0.16 | 0.41 | .681 |  | 0.06 | 0.14 | 0.43 | .670 |

*Note*. * Significant at *p* < .05, *** Significant at *p* < .001.
